# Cross-border collaboration between China and Myanmar for emergency response to imported vaccine derived poliovirus case

**DOI:** 10.1186/s12879-015-0745-y

**Published:** 2015-01-17

**Authors:** Hai-Bo Wang, Li-Fen Zhang, Wen-Zhou Yu, Ning Wen, Dong-Mei Yan, Jing-Jing Tang, Yong Zhang, Chun-Xiang Fan, Kathleen H Reilly, Wen-Bo Xu, Li Li, Zheng-Rong Ding, Hui-Ming Luo

**Affiliations:** Chinese Center for Disease Control and Prevention, 27 Nanwei Road, Xicheng District Beijing, 100050 PR China; Peking University Clinical Research Institute, Xueyuan Road 38#, Haidian District Beijing, 100191 PR China; Expanded Programme on Immunization, Yunnan Center for Disease Control and Prevention, 158 Dongsi Street, Kunming City, Yunnan province 650034 PR China; WHO WPRO Regional Polio Reference Laboratory, National Institute for Viral Disease Control and Prevention, Chinese Center for Disease Control and Prevention, 155 Changbai Road, Changping District Beijing, 102206 PR China; Independent Consultant, New York City, NY USA

**Keywords:** Vaccine derived poliovirus, Importation, Cross-border collaboration, China, Myanmar

## Abstract

**Background:**

This report describes emergency response following an imported vaccine derived poliovirus (VDPV) case from Myanmar to Yunnan Province, China and the cross-border collaboration between China and Myanmar. Immediately after confirmation of the VDPV case, China disseminated related information to Myanmar with the assistance of the World Health Organization.

**Methods:**

A series of epidemiological investigations were conducted, both in China and Myanmar, including retrospective searches of acute flaccid paralysis (AFP) cases, oral poliovirus vaccine (OPV) coverage assessment, and investigation of contacts and healthy children.

**Results:**

All children <2 years of age had not been vaccinated in the village where the VDPV case had lived in the past 2 years. Moreover, most areas were not covered for routine immunization in this township due to vaccine shortages and lack of operational funds for the past 2 years.

**Conclusions:**

Cross-border collaboration may have prevented a potential outbreak of VDPV in Myanmar. It is necessary to reinforce cross-border collaboration with neighboring countries in order to maximize the leverage of limited resources.

## Background

Since 2012, circulation of indigenous wild poliovirus (WPV) has been confined to three countries: Afghanistan, Nigeria, and Pakistan [1]. Despite significant achievements since the launch of the Global Polio Eradication Initiative in 1988, many previously polio-free countries remain at risk for the disease and have been affected by WPV importation from remaining endemic countries [2-5]. With increasing globalization, population mobility is contributing to the transmission of infectious diseases between countries, as cross-border population movement impedes efforts to prevent transmission of infectious diseases. For example, in 2009, 98.8% of total malaria cases in Yunnan Province, China were found to be imported from neighboring countries [6].

In China, the last indigenous case of WPV was reported in September 1994 [7]. China and the other countries of the Western Pacific Region were declared polio-free in October 2000 [8]. However, national health departments in China must remain vigilant regarding the risk of WPV importation from bordering endemic countries. Instances of WPV importation were detected in China before the region was declared polio-free: in 1995 and 1996 in Yunnan Province [9], and in 1999 in Qinghai province [10,11]. Moreover, after being polio-free for more than 10 years, on August 25, 2011, an outbreak was confirmed in Xinjiang Uygur Autonomous Region, China following importation of type 1 WPV originated from neighboring Pakistan [12-14].

Myanmar had no reported polio cases during the period of 2000-2005, but a single case of type 1 vaccine derived poliovirus (VDPV) was reported in April 2006. After several years without WPV cases, an outbreak of 11 type 1 WPV cases occurred in Myanmar in 2007, which was confirmed as the re-introduction of WPV, with the last reported paralyzed case in May 2007 [15]. Subsequently, an additional four type 1 VDPV cases were reported in 2007, and a type 1 VDPV case was reported in December 2010.

The Global Polio Eradication Initiative of World Health Organization (WHO) recommends reporting and laboratory testing of fecal specimens for all cases of acute flaccid paralysis (AFP) among children < 15 years of age and suspected poliomyelitis in a person of any age as the standard means of WPV and VDPV surveillance. The Chinese government initiated AFP surveillance in several provinces, following WHO guidelines in 1991. In 1993, surveillance was extended to the national level conducted by the Chinese Center for Disease Control and Prevention (China CDC) under the leadership of Ministry of Health (MoH). WHO established a global polio laboratory network, which includes a Polio Regional Reference Laboratory in China, to confirm poliovirus infections.

This report describes the cross-border collaboration between China and Myanmar on the public health response of an importation of VDPV case that occurred in Yunnan Province, China, in June 2012; the last reported case of WPV infection in Yunnan, China was imported from Myanmar in 1996 [9]. This cross-border collaboration and public health response is a model example for other countries which have borders with WPV endemic countries, and the lessons learned may be incorporated into national emergency response planning for a polio outbreak.

## Methods

### Setting and population

Yunnan Province is situated in southwest China, with an area of 394,000 km^2^. Yunnan shares 4,060 kilometers of international borders with Myanmar in the west and southwest and with Vietnam and Laos in the south. Six of 16 prefectures in Yunnan are adjacent with Myanmar, with nearly 2,000 kilometers of international borders. Moreover, there are few natural barriers between the two countries, and the border habitants of each country interact very frequently. In 2011 the population of Yunnan Province was estimated to be 46.3 million with 9.5 million children younger than 15 years of age; the average population density is 118 persons/km^2^, and the annual birth rate is approximately 12.7‰.

The VDPV case lived in a Village (Village A, Township B) which is a part of North Shan State in Myanmar with an estimated 73,000 population. Township B is under the leadership of Kokang Special Administrative Region bordering with Yunnan Province. Many parts of the township are geographically hard to reach and are unable to be accessed in the rainy season. The township has a 50-bed hospital in an urban area and one 16-bed station hospital in a sub-township. Medical treatment and public health services are provided by township health staff.

### Case ascertainment and VDPV case definition

Cases with paralytic polio were identified through an AFP surveillance system. CDC staff at the county-level routinely investigate AFP cases reported by health care centers and hospitals, collect stool specimens and assess residual paralysis 60 days after the onset of paralysis. Clinical and epidemiological information on AFP cases are abstracted from medical records and/or obtained from case investigations; a standard case investigation form is used in China.

In accordance with WHO current recommendations, a VDPV case was defined as an AFP case from whom Sabin-related poliovirus (type 2 with ≥6 nt VP1 substitutions; type1 and 3 with ≥10 nt VP1 substitutions) was isolated from ≥1 stool specimen without WPV isolated, and who was determined to be polio-compatible by a provincial Polio Expert Committee after the standard 60-days follow-up examination.

### Retrospective searching of AFP cases

Records of all county- and prefecture-level hospitals in Lincang City, Yunnan Province where the VDPV case sought health care were reviewed for additional AFP cases. In Myanmar, retrospective searches for AFP cases were conducted in hospitals in township B and through a house-to-house search in the affected village and neighboring areas. The definition of AFP case was AFP case <15 years old, or suspected polio case at any age, paralyzed between January 1, 2010 and the survey date.

### Oral poliovirus vaccine (OPV) coverage assessment

To determine immunization coverage, convenience surveys were conducted in four counties (two counties the case visited and two counties bordering Myanmar) in Yunnan Province. Quick assessment of OPV coverage was conducted and performance of routine immunization were also evaluated in Township B.

### Investigation of contacts and healthy children

Stool specimens were collected from hospital contacts of the VDPV case and healthy children <5 years of age in the two counties (Zhenkang County and Linxiang County) that the VDPV case visited in Lincang City, Yunnan Province. In Myanmar, stool specimens were also collected from close contacts of the VDPV case.

### Isolation and characterization of poliovirus isolates

Stool specimens were forwarded to the provincial polio laboratory where viral isolation was performed on L20B and RD cell cultures and viral isolates were identified by micro-neutralization assay. Poliovirus isolates were forwarded to the National Polio Laboratory where intratypic differentiation was performed by polymerase chain reaction–restriction fragment–length polymorphism and by enzyme-linked immunosorbent assay. The full VP1 genomic region of poliovirus isolates was sequenced with an ABI Prism BigDye Terminator Cycle Sequencing Ready Reaction kit and an automated DNA sequencer. Sequence data were compared with those of reference strains (GenBank accession no. AY184219). All contact specimens collected in Myanmar were sent to WHO accredited National Health Laboratory Yangon for poliovirus testing.

### Ethical considerations

This study was approved by the Chinese Center for Disease Control and Prevention institutional review board. Written informed consent regarding anonymizing publication of VDPV infection was provided by guardian of the index case in Myanmar and the guardians of four AFP cases in Lincang city of Yunnan Province, after study staff explained fully to guardian about the purpose of the study, and the risks and benefits of anonymizing publication.

### Statistical analysis

Statistical tests were performed using SAS 9.1 software (SAS Institute Inc, Cary, NC, USA). A *p*-value of <0.05 was considered the cut-off level for statistical significant for all analysis.

## Results

### VDPV case

The case, 18 months old, lived in a Village of Township B, North Shan State in Myanmar. The child had no previous history of OPV vaccination, no history of travelling outside the village 4-6 weeks prior to illness, or any family visits. In the first instance, the child developed high fever; two days later, the child was brought to a local clinic, where an intramuscular injection (drug was unknown) was given on his left buttock; fever subsided after injection, but the child developed bilateral weakness of the lower limbs on the same night; the weakness worsened by next day and the child was unable to stand or walk.

Four days later, the child went to a Hospital in Township B and was referred to a hospital in Zhenkang County (in China) where he was then referred to a Hospital in Lincang City and was reported as an AFP case. Type 1 VDPV strains were isolated from both stool specimens collected 6 and 7 days after the onset of AFP, with 21 (2.3%) nt VP1 substitutions from Sabin poliovirus. After staying in Lincang City for more than one week, the child returned to Myanmar because of no improvement in symptoms. Therefore, no additional stool sample was collected and VDPV excretion was not followed up for the case. No medical test for immunodeficiency was done without final classification of VDPV.

### Cross-border collaboration between China and Myanmar—information sharing

Immediately after confirmation of the VDPV case, China CDC informed WHO about the VDPV case and required the assistance of WHO for sharing related information with Myanmar. The government of Myanmar received the report about the VDPV importation with the help of the WHO country office in China, WHO Western Pacific Regional Office, WHO Regional Office for South-East Asia, and WHO country office in Myanmar.

Based on the information provided by the Chinese MoH, Myanmar’s MoH, and the WHO country office in Myanmar, the VDPV case was located and additional detailed epidemiological information was collected on the outbreak and immunization activities were conducted including OPV coverage assessment, AFP case searching and specimen collection among close contacts and healthy children by a joint team consisting of officials from Myanmar’s MoH and WHO country office in Myanmar. China, WHO, and Myanmar maintained communication regarding the epidemiology of the outbreak and emergency response activities.

### Investigation of contacts and healthy children

In Yunnan Province, stool specimens were collected from two hospital contacts who stayed in the same room in the hospital of Lincang City, with negative results for both poliovirus and non-polio enterovirus (NPEV). In Linxiang and Zhenkang counties, stool specimens were also collected from 50 healthy children <5 years of age in an area with a concentrated migrant population, all of which were negative for poliovirus except 8 (16%) specimens positive for NPEV.

In Myanmar, stool specimens were collected from 10 close contacts including family and those who encountered the VDPV case in the hospital after paralysis (4 in the Village where the case lived, 1 in another Village where the case had stayed and 5 in Township Hospital), with negative results for both poliovirus and NPEV.

### OPV coverage assessment and routine immunization

Convenience surveys for OPV coverage were conducted in 4 counties of Lincang City: Linxiang and Zhenkang Counties that the case visited, and two other counties (Gengma and Cangyuan) which border Myanmar. A total of 365 children were enrolled: 65 < 1 year of age and 300 ≥ 1 year of age (Table 1). Among the 365 subjects enrolled, 356 (97.5%) had a vaccination certificate and 363 (99.5%) had received at least one dose of OPV. Among 300 participants ≥1 year of age, 279 (93.0) had received more than 3 doses of OPV (OPV_3_). OPV_3_ rates by county were also high among subjects ≥1 year of age, with the lowest rate (86.3%) in market place of Linxiang County.

**Table 1:** **Result of OPV coverage assessment in 4 counties of Lincang City, Yunnan Province**

**County**	**Investigation site**	**Age Group (Months)**	**No. surveyed**	**Vaccination certificate**			**OPV immunization history (dose)**					**OPV Vaccination Rate (%)**	**OPV Fully Vaccination Rate (%)**
				**Yes**	**No**	**Rate (%)**	**0**	**1**	**2**	**≥3**	**Unknown**		
Linxiang	Market Place	2-12	18	18	0	100.0	0	3	1	14	0	100.0	77.8
		>12	80	80	0	100.0	0	3	8	69	0	100.0	86.3
Zhenkang	Market Place	2-12	5	4	1	80.0	1	1	1	2	0	80.0	40.0
		>12	25	24	1	96.0	0	0	1	24	0	100.0	96.0
	Mengpeng Township	2-12	6	6	0	100.0	0	1	2	3	0	100.0	50.0
		>12	27	26	1	96.3	0	0	3	24	0	100.0	88.9
	Mengdui Township	2-12	6	6	0	100.0	0	3	0	3	0	100.0	50.0
		>12	24	24	0	100.0	0	0	0	24	0	100.0	100.0
Cangyuan	Market Place	2-12	12	10	2	83.3	0	1	1	10	0	100.0	83.3
		>12	49	46	3	93.9	0	1	0	48	0	100.0	98.0
	Mengjiao Town	2-12	4	4	0	100.0	1	0	0	3	0	75.0	75.0
		>12	26	26	0	100.0	0	1	0	25	0	100.0	96.2
Gengma	Market Place	2-12	6	6	0	100.0	0	0	0	6	0	100.0	100.0
		>12	23	23	0	100.0	0	1	1	21	0	100.0	91.3
	Hepai Town	2-12	1	1	0	100.0	0	0	0	1	0	100.0	100.0
		>12	28	28	0	100.0	0	0	0	28	0	100.0	100.0
	Mengding Town	2-12	7	6	1	85.7	0	2	2	3	0	100.0	42.9
		>12	18	18	0	100.0	0	1	1	16	0	100.0	88.9
Total		2-12	65	61	4	93.8	2	11	7	45	0	96.9	69.2
		>12	300	295	5	98.3	0	7	14	279	0	100.0	93.0

The VDPV case had a three month old sibling who had also not received any vaccinations. The township medical officer reported that there had been no vaccination conducted since last 2 years in this village, and that most villages did not have adequate access to vaccination in Township B. Moreover, most areas were not covered for routine immunization in this township due to vaccine shortages and lack of operational funds in the past 2 years. The reported OPV_3_ coverage among children <1 year of age were 48% in 2009, 63% in 2010, and 73% in 2011, respectively.

### Retrospective searching of AFP cases

Medical records for both inpatients and outpatients were reviewed in 9 hospitals at the county level and above in the 4 aforementioned counties of Lincang City. A total of 4 AFP cases were found: 2 cases in Linxiang County, one in Zhenkang County, and one in Cangyuan County; all of these cases had been reported.

In Myanmar, no additional AFP cases were found during active case searches in Township B Hospital nor during house-to-house searches in the Village and neighboring areas.

### AFP surveillance performance

The overall annualized incidence of non-polio AFP (NPAFP) was 2.98 per 100,000 children < 15 years of age in Yunnan Province from January to May 2012, and NPAFP rates were >1/100,000 in almost all prefectures except for Xishuangbanna Prefecture and Nujiang City where no NPAFP cases were reported (Table 2). In 2011, with the exception of Xishuangbanna Prefecture, NPAFP rates were higher than 1 case per 100,000 children in all prefectures. Another important indicator, adequate stool specimen collection rate, was also met in almost all prefectures.

**Table 2:** **Main performance indicators of AFP surveillance among children <15 years of age by prefecture in Yunnan Province, 2011 and 2012 (January to May)**

**Time period**	**Prefecture**	**No. of NPAFP cases**	**Annualized NPAFP incidence (1/100, 000)** ^**§**^	**Investigation within 48 hours (%)**	**Two stool samples collected (%)**	**Adequate stool sample collected (%)**	**Stool samples delivered within 7 days (%)**	**Lab result reported within 28 days (%)**
2011	Kunming City	22	1.71	100.0	81.8	72.7	100.0	100.0
	Qujing City	21	1.54	95.2	90.5	90.5	100.0	100.0
	Yuxi City	31	6.53	96.8	96.8	96.8	100.0	100.0
	Baoshan City	20	3.69	100.0	90.0	90.0	100.0	100.0
	Zhaotong City	22	1.84	90.9	95.5	90.9	90.9	100.0
	Chuxiong Prefecture	22	3.93	100.0	100.0	100.0	100.0	100.0
	Honghe Prefecture	20	2.08	100.0	85.0	85.0	100.0	95.0
	Wenshan Prefecture	19	2.28	100.0	89.5	84.2	88.9	100.0
	Pu’er City	16	2.86	100.0	93.8	93.8	100.0	100.0
	Xishuangbanna Prefecture	2	0.87	100.0	50.0	50.0	100.0	100.0
	Dali Prefecture	18	2.35	94.4	100.0	100.0	100.0	100.0
	Dehong Prefecture	17	6.40	100.0	100.0	100.0	100.0	100.0
	Lijiang City	12	4.52	100.0	83.3	83.3	100.0	100.0
	Nujiang City	4	3.38	100.0	50.0	50.0	100.0	100.0
	Diqing City	1	1.19	100.0	100.0	100	100.0	100.0
	Lincang City	13	2.32	100.0	92.3	92.3	92.3	100.0
	Yunnan Province	260	2.58	98.1	91.5	90.0	98.1	99.6
2012 (January to May)	Kunming City	20	4.89	100.0	95.0	95.0	100.0	100.0
	Qujing City	8	1.41	100.0	75.0	75.0	100.0	100.0
	Yuxi City	4	2.27	100.0	100.0	100.0	100.0	100.0
	Baoshan City	4	1.89	100.0	100.0	100.0	100.0	100.0
	Zhaotong City	14	2.44	100.0	92.9	92.9	78.6	100.0
	Chuxiong Prefecture	7	3.59	100.0	100.0	100.0	100.0	100.0
	Honghe Prefecture	10	2.51	100.0	100.0	100.0	100.0	100.0
	Wenshan Prefecture	5	1.44	100.0	100.0	100.0	100.0	100.0
	Pu’er City	7	3.60	100.0	85.7	85.7	71.4	100.0
	Xishuangbanna Prefecture	0	0.0	−	−	−	−	−
	Dali Prefecture	9	3.23	100.0	100.0	100.0	100.0	100.0
	Dehong Prefecture	11	10.49	100.0	100.0	100.0	90.9	100.0
	Lijiang City	8	8.58	100.0	100.0	100.0	100.0	100.0
	Nujiang City	0	0.0	−	−	−	−	−
	Diqing City	1	3.30	100.0	100.0	100.0	100.0	100.0
	Lincang City	9	4.38	100.0	100.0	100.0	100.0	100.0
	Yunnan Province	117	2.98	100.0	95.7	95.7	94.9	100.0

^§^Annualized NPAFP incidence in 2012 (January to May), No. of AFP Cases × 12/5 × 100, 000/100, 000)/ No. of children <15 years of age.

In Township B Hospital, doctors and health workers had fairly good knowledge on AFP surveillance. An AFP poster for awareness purpose, including almost all the diseases with AFP symptom, was displayed in township hospital premises. However, there were many Chinese clinics in Township B, which were not part of the AFP surveillance system. AFP cases were not reported in Township B and neighboring townships, and the sensitivity indicator (>1 case per 100,000 children <15 years of age) of AFP surveillance was not met.

### Supplementary immunization activities (SIAs)

OPV catch-ups were conducted among children <6 years of age in Lincang City, Yunnan Province. The targeted children also included those from Myanmar who attend school, travel or live in Lincang City. OPV catch-ups in Yunnan Province and SIAs in Myanmar were conducted at similar time.

In order to control the outbreak timely and effectively, outbreak response immunization was conducted in a month after paralysis onset of the VDPV case in 6 neighboring villages, including one urban border crossing ward of Township B. Six hundred fifty children were given one dose of trivalent OPV. In addition, two months later, two rounds of house-to-house supplementary immunization activities (SIAs) were conducted in 19 townships of North Shan State in Myanmar, covering 233,000 children <6 years of age.

## Discussion

Based on clinical examination the index child was diagnosed as clinically compatible with polio, and type 1 VDPV (21 nucleotides substitution) was isolated from stool specimens, so the child was classified as a VDPV case. This is the first report of an imported VDPV case in China, although WPV importation had been detected by AFP surveillance in the past. The Chinese MOH immediately disseminated related epidemiological information to Myanmar upon detection of the VDPV case with assistance from WHO. Based on information provided by the Chinese MoH, a timely emergency response was conducted in Myanmar, including an epidemiological investigation and SIAs. China and Myanmar maintained communication, regularly updating each other on investigation activities. This effort should be a model example of cross-border collaboration for global polio eradication.

High quality of routine immunization against polio and AFP surveillance are two key elements for polio eradication. Low immunization immunity due to poor OPV coverage in routine immunization was the major risk factor related with VDPV outbreak. The township medical officer reported that most areas were not covered for routine immunization in this township in the past 2 years. The reported OPV_3_ coverage among children <1 year of age were 48% in 2009, 63% in 2010, and 73% in 2011, respectively. Moreover, AFP Surveillance system was found to be inadequate in the area of the VDPV case. Therefore, retrospective searching of AFP cases was necessary for identifying under-reported AFP cases during field investigation.

Imported WPV and VDPV cases were detected timely by AFP surveillance system in Yunnan Province, which was based on the performance of AFP surveillance. Moreover, high population immunity had prevented transmission of poliovirus without subsequent outbreaks, although importation has occurred many times. The quick assessment of OPV coverage showed that the immunization rate was high in Lincang City, with 93.0% of children >1 year of age fully immunized. A serologic study conducted to estimate poliovirus antibody seroprevalence in 2010, indicated that more than 85% of children were seropositive for poliovirus serotype 1 in Lincang City [16]. The risk of cVDPV outbreak was considered to be low according to risk assessment, so OPV catch-up other than SIAs was conducted. It is necessary to strengthen routine immunization and AFP surveillance further until WPV is eradicated worldwide.

OPV is recommended by national immunization program in both China and Myanmar. However, due to a high liability of genetic mutation and recombination with other enteroviruses [17], one disadvantage associated with OPV is the rare occurrence of VDPVs. The cVDPV in Nigeria have indicated the potential risk of prolonged transmission of the vaccine virus among population with non-optimal immunity [18]. The goal of polio eradication is to complete eradication and containment of all wild, vaccine-related and Sabin polioviruses. Until WPV can be eliminated then programs utilizing OPV must be continued which entails an ongoing risk of VDPV outbreaks. Hence efforts to eliminate WPV must continue, and all countries must maintain effective immunization and AFP surveillance programs.

Timely information sharing between China and Myanmar may have avoided a potential outbreak of circulated VDPV (cVDPV) in Myanmar, considering the immunity gap among children in this high risk area where the VDPV case lived. The success in collaboration and the potential benefits attained emphasize the need to strengthen cross-border collaborative efforts among countries that neighbor endemic countries. It is noteworthy that increasing efforts are taken for cross-border collaboration in recent years. Intense cross-border migration continues in both directions, favoring continuous virus transmission between Afghan and Pakistan [19]. Therefore, synchronized cross-border polio campaigns were conducted in these two countries, ensuring simultaneous and comprehensive coverage of children in transit through the border areas [20]. In addition, many of cross-border meetings were held to explore a formalized approach to collaboration [21,22].

However, it took over 7 days for Myanmar to receive the information on the VDPV case, which was due to many counterparts involved in the information delivery in addition to the countries of China and Myanmar, which included the WHO country office in China, WHO Western Pacific Regional Office, WHO Regional Office for South-East Asia and WHO country office in Myanmar. To promote further efficient communication, working mechanisms for information exchange and emergency response collaboration should be developed between China and Myanmar. Historically, to control WPV importation from Myanmar, China and Myanmar simultaneously conducted OPV SIAs in the border areas of Yunnan Province, China and Shan and Kachin States, Myanmar. Due to good collaboration, a WPV outbreak did not take place in Yunnan Province, although imported WPV cases were monitored [9]. Cross-border collaboration with neighboring countries is particularly important for China, as it shares a border with two endemic countries (Afghanistan and Pakistan). However, a formalized collaboration approach is lacking. Based on the previous experience collaborating with Myanmar, the following mechanisms should be considered:

- A list of key contacts should be developed, who are responsible for polio-related issues on each side of the border;
- AFP surveillance and coverage data should be shared regularly;
- Each country should immediately inform the other upon detecting WPV cases, VDPV cases or a cluster of AFP cases in border townships or counties;
- Cross-border meetings should be conducted regularly to better understand each other public health system;
- Personnel and resources should be shared, including technical guidance; and
- SIAs should be synchronized if a WPV outbreak is detected.

## Conclusion

The collaboration between China and Myanmar may have prevented a potential cVDPV outbreak in Myanmar. Considering China shares borders with two endemic countries, it is necessary to reinforce cross-border collaboration with neighboring countries, which can maximize the leverage of limited resources. Conducting high quality routine immunization and AFP surveillance should be maintained until WPV is eradicated worldwide.
